# Investigating the Utility of Leukocyte Sialic Acid Measurements in Lysosomal Free Sialic Acid Storage Disorder

**DOI:** 10.1002/jmd2.70029

**Published:** 2025-06-16

**Authors:** Marya S. Sabir, Laura Pollard, Lynne Wolfe, David R. Adams, Carla Ciccone, Petcharat Leoyklang, Frances M. Platt, Marjan Huizing, William A. Gahl, May Christine V. Malicdan

**Affiliations:** ^1^ NIH Undiagnosed Diseases Program, National Human Genome Research Institute, National Institutes of Health Bethesda Maryland USA; ^2^ NIH Oxford‐Cambridge Scholars Program University of Oxford Oxford UK; ^3^ Biochemical Genetics Laboratory Greenwood Genetic Center Greenwood South Carolina USA; ^4^ Office of the Clinical Director, National Human Genome Research Institute, National Institutes of Health Bethesda Maryland USA; ^5^ Human Biochemical Genetics Section, Medical Genetics Branch, National Human Genome Research Institute, National Institutes of Health Bethesda Maryland USA; ^6^ Department of Pharmacology University of Oxford Oxford UK

**Keywords:** leukocytes, lysosomal storage disorders, Salla disease, sialic acid, sialin, SLC17A5

## Abstract

Lysosomal free sialic acid storage disorder (FSASD) is a rare, multisystem neurodegenerative disease caused by biallelic pathogenic variants in *SLC17A5*, encoding sialin. FSASD is characterized by aberrant accumulation of unconjugated “free” sialic acid (Neu5Ac) within lysosomes. Depending on the specific genetic variants, affected individuals may present with either a rapidly fatal disease or progressive neurodegeneration. While skin fibroblasts have traditionally been used for diagnosis and research, the use of leukocytes in FSASD remains underexplored. This study examined Neu5Ac levels in leukocytes from three individuals with FSASD carrying distinct *SLC17A5* variants. The levels in affected individuals were compared to three different groups: (1) the unaffected biological parents of each case; (2) subjects for whom 14 distinct lysosomal storage disorders (LSDs) were excluded based on enzyme analysis (*n* = 11); and (3) participants with a confirmed LSD diagnosis, as determined by enzyme analysis (*n* = 9). Individuals with FSASD exhibited significantly higher levels of free Neu5Ac compared to their unaffected biological parents (36‐fold), LSD‐negative subjects (22‐fold), and individuals with other LSDs (49‐fold). Although total Neu5Ac levels showed a non‐significant trend toward an increase in FSASD (1.3‐fold), this was primarily due to elevated free Neu5Ac, as bound Neu5Ac was slightly decreased in the leukocytes of FSASD cases relative to their unaffected parents. Overall, these findings highlight leukocytes as a valuable, minimally invasive cellular model for FSASD, offering an alternative, reliable diagnostic tool and a potential platform for monitoring therapeutic responses in future intervention trials.

## Graphical abstract

Schematic illustrating the study design and key findings. Figure created in Biorender.com.

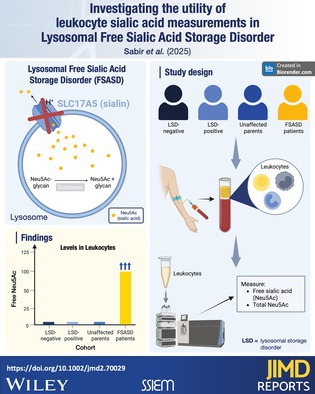

**Summary**

- This study demonstrates that leukocytes from individuals with FSASD exhibit significantly elevated free sialic acid levels, indicating their potential as a useful cellular model and a reliable biomarker for diagnosis and monitoring therapeutic outcomes in future intervention trials.

## 1 Introduction

Lysosomal free sialic acid storage disorder (FSASD) is a rare, multisystem neurodegenerative disorder caused by biallelic pathogenic variants in *SLC17A5* [1, 2]. *SLC17A5* encodes sialin, a proton‐coupled transporter responsible for the efflux of sialic acid (*N*‐acetylneuraminic acid; Neu5Ac) and other acidic sugars from lysosomes to the cytosol [3, 4, 5, 6]. Defective sialin activity results in excess accumulation of unconjugated (“free”) Neu5Ac within lysosomes, giving rise to a spectrum of clinical presentations.

Globally, approximately 250 individuals with FSASD have been reported with biallelic variants in *SLC17A5*. Among these, around 185 (75%) harbor the Finnish founder missense variant, *SLC17A5* c.115C>T (p.Arg39Cys), in either a homozygous or compound heterozygous state, while nearly 65 individuals possess other biallelic variants [7, 8, 9]. FSASD encompasses three clinical subtypes: infantile FSASD (MIM#269920), intermediate severe FSASD, and mild FSASD (MIM#604369; also known as Salla disease and characterized by homozygosity for the p.Arg39Cys variant) [2, 9]. Individuals with mild FSASD typically appear normal at birth and do not exhibit overt neurological symptoms; however, over time, they develop progressive neurological deterioration, characterized by mild‐to‐moderate psychomotor delays, spasticity, athetosis, and seizures [2]. In contrast, individuals with infantile FSASD present shortly after birth with severe developmental delays, coarse facial features, hepatosplenomegaly, and cardiomegaly, leading to early childhood mortality [2]. Individuals with FSASD excrete 10–100 times the normal levels of free Neu5Ac in urine [9], highlighting its potential as a fluid biomarker for the disorder. Of note, elevated urinary free sialic acid levels can also occur in other conditions, such as sialuria (increased by 100–1000 fold) [10, 11, 12] and *N*‐acetylneuraminate pyruvate lyase deficiency (increased by approximately 10‐fold) [13].

Skin‐derived fibroblasts from individuals affected with FSASD have been extensively used to model the disorder and derive mechanistic insights [14, 15]. Importantly, cultured fibroblasts display elevated levels of free Neu5Ac, both in lysosome‐enriched fractions as well as whole‐cell lysates [16, 17, 18, 19, 20]. In contrast, reports of unconjugated Neu5Ac concentrations in leukocytes are limited. Mancini and colleagues (1992) observed significant elevation (10‐ to 30‐fold) of free Neu5Ac in peripheral blood total leukocytes as well as lymphocyte and granulocyte subpopulations in an affected individual of Dutch ancestry [19]. Additionally, Baumkotter et al. (1985) reported mildly elevated (2.4‐fold) free Neu5Ac levels in leukocytes of a 4‐year‐old male with FSASD [20].

In this study, we aimed to profile the levels of Neu5Ac in peripheral blood leukocytes derived from three individuals with FSASD carrying distinct *SLC17A5* variants. We also measured Neu5Ac levels in the biological parents of each affected individual to determine the levels in obligate heterozygotes. To demonstrate specificity, we compared these results with those from individuals affected by other lysosomal storage disorders, as well as with individuals for whom 14 lysosomal storage disorders were excluded through enzyme analysis.

## 2 Materials and Methods

### 2.1 Study Populations

Whole blood samples were collected as part of the NIH FSASD Pilot Natural History Study under the Congenital Disorders of Glycosylation protocol (14‐HG‐0071; NCT04199000), approved by the NHGRI Institutional Review Board. This study includes the *SLC17A5* variant information and urinary sialic acid levels from three probands with FSASD, who are also part of a separate manuscript on the natural history of the condition, currently in preparation. Briefly, one female and two male participants diagnosed with FSASD were recruited to the National Institutes of Health (Bethesda, MD, USA) for clinical assessment and biospecimen collection. These individuals had intermediate severe FSASD, each carrying different pairs of *SLC17A5* variants, and ranging in age from 3.7 to 6.1 years at the time of sample collection (Table 1), and represented mixed racial backgrounds and included those of Hispanic/Latino ethnicity (Table 1). The biological parents of each affected individual were also consented and provided blood samples for research purposes.

**TABLE 1:** FSASD cohort demographics, disease classification, and *SLC17A5* variants.

Individual with FSASD no.	Sex	Age at sample collection (years)	Race/ethnicity	FSASD disease classification	*SLC17A5* variants
1	F	4.8	Multiple race/Hispanic or Latino	Intermediate severe	c.406A>G p.(Lys136Glu) and c.533delC p.(Thr178Asnfs*34)
2	M	3.7	Multiple race/Hispanic or Latino	Intermediate severe	c.291G>A p.(Thr97=) and c.918T>G p.(Tyr306*)
3	M	6.1	Multiple race/Hispanic or Latino	Intermediate severe	c.115C>T p.(Arg39Cys) and c.431T>G p.(Leu144Arg)

*Note: SLC17A5* nucleotide substitution reference is NM_012434.5 and amino acid substitution reference is NP_036566.

Abbreviations: F, female; M, male; FSASD, free sialic acid storage disorder; *, stop codon.

The LSD‐negative group (*n* = 11) included deidentified samples tested by the Greenwood Genetic Center (GGC; South Carolina, USA) that exhibited normal levels of lysosomal enzyme activities that ruled out a diagnosis of 14 different lysosomal storage disorders (LSDs; https://ggc.org/test‐finder‐item/lysosomal‐storage‐disease‐enzyme‐panel). In contrast, the LSD group (*n* = 9) consists of deidentified samples from individuals affected with one of nine different LSDs. The LSD group included individuals with the following disorders: Fabry disease, Gaucher disease, mucopolysaccharidosis (MPS) type I, GM1 gangliosidosis, MPS type IIIC, MPS type IIIA, metachromatic leukodystrophy, alpha‐mannosidosis, and Pompe disease. The LSD group consisted of five females and four males, aged 1 month to 68 years at the time of screening (Table S1). The LSD‐negative group comprised six females and five males, with ages ranging from 4 months to 56 years (Table S1).

### 2.2 Isolation of Peripheral Blood Leukocytes

Whole blood samples were collected in tubes containing sodium heparin as the anticoagulant. Briefly, leukocytes were isolated using 3% dextran sedimentation, followed by multiple saline washes to ensure purity.

### 2.3 Quantification of Sialic Acid in Leukocytes and Urine

Neu5Ac, the most abundant mammalian sialic acid [21], is referred to as “sialic acid” in this report. Briefly, isolated leukocytes or fibroblasts were sonicated in 1 mL water, and 25 μL of sonicate was mixed with 25 μL 21 μM 1,2,3‐^13^C_3_‐*N*‐acetyl‐*D*‐neuraminic acid internal standard and either 100 μL HPLC‐grade water (free sialic acid) or 100 μL 63 mM sulfuric acid (total sialic acid). Total sialic acid samples were incubated for 1 h at 80°C. Only free (not total) sialic acid samples were filtered using a Spin‐X 0.22‐mm microcentrifuge filter tube. Total and free sialic acid were analyzed by UPLC‐MS/MS (Waters Acquity I‐Class; Xevo TQ‐S MS/MS), using a C18 reverse‐phase column (Waters Acquity UPLC HSS T3) for chromatographic separation with a gradient between 100% 0.05 M ammonium formate buffer, pH 3.0, and 100% acetonitrile with a 3‐min injection time. Tandem mass spectrometry was operated in selected reaction monitoring mode (electrospray ionization, negative ion mode) for detection with mass transitions of 308.2 > 87 for sialic acid and 311.2 > 90 for the internal standard. Quantification was accomplished by stable isotope dilution and comparison to a standard curve containing 10 sialic acid concentrations ranging from 0.1 to 100 μM. All sample runs demonstrated calibration curves with linearity exceeding *r* ^2^ > 0.98. Sialic acid concentrations were normalized by total protein concentration as determined by the Lowry method.

Free and total sialic acid levels in urine were measured by high performance liquid chromatography (HPLC). 1,2‐Di‐amino‐4,5methylenedioxybenzene dihydrochloride (DMB) reacts with sialic acid to form a fluorescent complex. For total sialic acid, 200 μL 0.1 N sulfuric acid was added to 5 μL urine and incubated for 3 h at 80°C. For free sialic acid, 200 μL 0.1 N sulfuric acid was added to 5 μL urine immediately prior to adding 200 μL DMB solution to both free sialic acid and total sialic acid samples. All samples were then incubated in the dark for 2.5 h at 50°C. HPLC (5 μL injection volume, 0.9 mL/min flow rate of buffer consisting of 90 mL acetonitrile, 70 mL methanol, 840 mL water) was used to separate sialic acid from underivatized DMB, with fluorescence detection (excitation wavelength 373 nm, emission wavelength 448 nm). Quantification was achieved using a standard curve with five calibrator concentrations ranging from 50 to 1000 μM. All sample runs demonstrated calibration curves with linearity exceeding *r* ^2^ > 0.98. Sialic acid concentrations were normalized by creatinine concentration, as determined by the Jaffé reaction (alkaline picric acid).

Bound sialic acid concentrations were calculated by subtracting the measured levels of free sialic acid from the corresponding total sialic acid concentrations.

### 2.4 Statistical Analyses

Statistical analyses were conducted using an unpaired *t*‐test or ordinary one‐way ANOVA between FSASD and comparison samples using GraphPad Prism (GraphPad Software, version 10.0.3). A two‐sided *p*‐value significance was set at 0.05.

## 3 Results

We measured free and total sialic acid levels in peripheral blood leukocytes from three individuals with FSASD, each carrying distinct pathogenic *SLC17A5* variant pairs. One female had a missense and a nonsense variant, one male possessed a missense and a splice site variant, and another male harbored two missense variants (Table 1). The levels in affected individuals were then compared to: (1) each case's biological parents (*n* = 6); (2) subjects without 14 known lysosomal storage disorders (LSD; *n* = 11); and (3) participants with a LSD diagnosis (*n* = 9).

**FIGURE 1:**
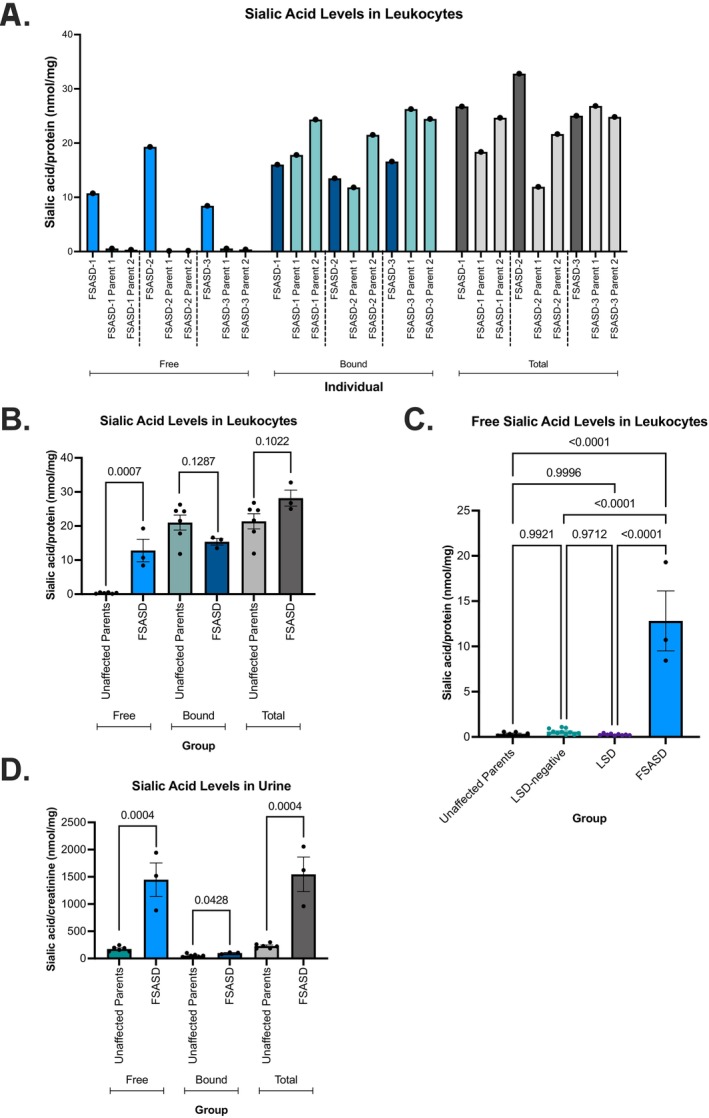
Sialic acid levels in FSASD and unaffected individuals. (A) Free, bound, and total sialic acid levels in leukocytes. (B) Free, bound, and total levels of sialic acid in leukocytes per group. (C) Comparison of free sialic acid levels in leukocytes from FSASD cases versus LSD‐negative and LSD cases. (D) Free, bound, and total levels of sialic acid in urine. Leukocyte levels were normalized to protein concentration and urine levels were normalized to creatinine. Mean ± SEM, unpaired *t*‐test or ordinary one‐way ANOVA with *p*‐values as indicated. LSD, lysosomal storage disorder.

Peripheral blood leukocytes derived from individuals with FSASD exhibited significantly elevated levels of free sialic acid (12.81 ± 3.31 nmol/mg protein) compared to the unaffected parental group (0.349 ± 0.079 nmol/mg protein), representing a 36‐fold increase in FSASD (Figure 1A,B). Total sialic levels displayed a non‐significant (*p* = 0.1022) upward trend in FSASD (28.19 ± 2.36 nmol/mg protein) compared to the parental group (21.38 ± 2.24 nmol/mg protein), demonstrating a 1.3‐fold increase in FSASD (Figure 1A,B). The elevated total sialic acid levels in FSASD primarily reflect an increase in free sialic acid, rather than bound sialic acid, which are non‐significantly (*p* = 0.1287) reduced in FSASD (15.38 ± 0.95 nmol/mg protein) compared to the parental group (21.03 ± 2.20 nmol/mg protein) (Figure 1A,B).

Leukocytes derived from individuals with FSASD displayed significantly increased levels of free sialic acid (12.81 ± 3.31 nmol/mg protein) compared to the LSD‐negative group (0.582 ± 0.092) nmol/mg protein), corresponding to a 22‐fold increase in FSASD (Figure 1C). FSASD leukocytes also exhibited a marked elevation in free sialic acid levels, nearly 49 times higher than those observed in the LSD group (0.26 ± 0.031 nmol/mg protein), as shown in Figure 1C. Free sialic acid levels were comparable across the unaffected parents, LSD‐negative, and LSD groups, and no significant differences were observed between them (Figure 1C).

Free sialic acid levels in urine samples from individuals with FSASD were significantly elevated (1447 ± 308.3 nmol/mg creatinine) compared to the unaffected parental group (174.2 ± 18.79 nmol/mg creatinine), indicating an 8.3‐fold increase in FSASD (Figure 1D). Similarly, total sialic acid levels were elevated in FSASD (1546 ± 318.2 nmol/mg creatinine) compared to the parental group (228.2 ± 18.35 nmol/mg creatinine), reflecting a 6.8‐fold increase in FSASD (Figure 1D). These changes in total sialic acid content are directly attributed to elevated free sialic acid, as only a borderline significant difference was observed in bound sialic acid levels between FSASD and parental subjects (*p* = 0.043) (Figure 1D). Figure S3 illustrates the comparison of free and total sialic acid levels in individuals with FSASD to age‐dependent reference values, showing that all individuals with FSASD exhibit elevated levels compared to the reference range.

Exploratory analyses revealed no significant correlation between free sialic acid levels in leukocytes and urine; however, this finding is limited by small sample size (*n* = 3 FSASD cases) (Figure S1). Additionally, leukocyte free sialic acid concentrations did not correlate with age in LSD‐negative and LSD control groups (Figure S2; *r* = 0.1173, *p* = 0.6225), indicating that age is not a confounding variable in this cohort. Free and total sialic acid levels in fibroblasts were also quantified in affected individual #1 (FSASD‐1, as shown in Table 1), revealing a significant increase when compared to two external unaffected participants (3.2‐fold, *p* = 0.0238) (Figure S4). For comparative analysis, free, bound, and total sialic acid levels were also measured in a fibroblast line from an additional individual with intermediate severe FSASD, as well as in fibroblasts from three individuals homozygous for the common p.Arg39Cys variant associated with Salla disease (Figure S4). These data collectively demonstrated elevated free sialic acid levels in fibroblasts derived from individuals with intermediate severe FSASD compared to those with attenuated disease.

## 4 Discussion and Limitations

Cellular models for studying lysosomal free sialic acid storage disorder have been historically limited to skin‐derived fibroblasts and, to a significantly lesser extent, leukocytes from affected individuals. The collection and sampling of fibroblasts is more invasive and labor‐intensive than that of peripheral blood leukocytes and does not accurately reflect the physiological context, which may limit its suitability for longitudinal clinical monitoring. This study aimed to evaluate the potential of leukocytes as a useful model for FSASD.

Individuals with FSASD displayed a 36‐fold increase in free sialic acid levels in peripheral blood leukocytes compared to their biological parents, who are obligate heterozygotes. FSASD cases exhibited 22‐ and 49‐fold increases in free Neu5Ac levels relative to LSD‐negative and other LSD cases, respectively. Although absolute levels of free and total Neu5Ac were higher in urine than in whole‐cell lysates of leukocytes, the fold change in urine was lower, and urinary levels represent a *non‐cellular* fluid biomarker. The elevated free Neu5Ac levels in leukocytes may reflect systemic accumulation in other tissues. Interestingly, in FSASD‐1, free Neu5Ac levels in leukocytes were 18.4‐fold higher than in the LSD‐negative reference group, compared to a 3.2‐fold increase in fibroblasts from the same individual. It is important to note that this discrepancy may stem from differences in lysosome abundance between cell types.

Our findings suggest that hypersialylation of glycans, a feature observed in cancer and other conditions [22, 23], is unlikely to play a significant role in FSASD, as conjugated (“bound”) sialic acid levels—calculated by subtracting free Neu5Ac from total Neu5Ac—were modestly reduced and not statistically significant in FSASD.

This study has limitations. First, the FSASD cohort was small (*n* = 3 cases) and did not include mild FSASD cases (homozygous for the common p.Arg39Cys variant) or severe cases (carrying biallelic severe *SLC17A5* variants), limiting our ability to assess Neu5Ac levels across the full clinical spectrum. Another limitation of this study is the lack of age‐matched controls with urinary free sialic acid measurements; although unaffected parents were used to account for shared genetic background, their older age may confound the magnitude of observed differences. Next, Neu5Ac levels were measured at a single timepoint, underscoring the need for longitudinal studies to evaluate these markers as potential indicators of disease progression. Furthermore, the study did not profile Neu5Ac levels in leukocyte subpopulations, such as granulocytes and lymphocytes, limiting the granularity of the findings. Changes in leukocyte subpopulations, possibly driven by factors such as active infection(s), may also influence overall Neu5Ac levels. Next, we measured sialic acid levels in whole‐cell lysates, whereas many previous studies have analyzed sialic acid levels in lysosome‐enriched fractions, typically using fibroblasts [16, 17, 18]. Consequently, direct comparison of our findings in leukocytes with those from earlier cellular studies is challenging, except for [19, 20], which also utilized whole‐cell lysates of leukocytes. While whole‐cell extracts are informative and less time‐consuming, requiring significantly fewer specialized procedures than lysosomal fractionation, an important question remains whether lysosomal fractions from leukocytes may reveal even higher levels of unconjugated Neu5Ac. Lastly, while elevated leukocyte free sialic acid may serve as a marker for FSASD, related sialic acid metabolism disorders including sialuria and *N*‐acetylneuraminate pyruvate lyase deficiency may exhibit similar biochemical abnormalities, although leukocyte free sialic acid levels in these conditions have not been reported. Therefore, molecular confirmation remains essential for diagnostic specificity.

Overall, this study supports the use of leukocyte‐based Neu5Ac measurement as a promising diagnostic and monitoring tool for FSASD. This less invasive method could facilitate longitudinal assessments, particularly in mild FSASD cases, and may prove valuable for evaluating therapeutic responses in future clinical trials.

## Author Contributions

M.S.S., M.H., and M.C.V.M. contributed to the conceptualization of the study. L.P. and P.L. performed the investigation of leukocytes and fibroblasts, respectively. Formal analysis and data visualization were conducted by M.S.S. Project administration was managed by M.S.S. and M.C.V.M. Resources were provided by L.P., L.W., D.R.A., and C.C. The study was supervised by F.M.P., M.H., W.A.G., and M.C.V.M. M.S.S. prepared the original draft, and all authors (M.S.S., L.P., L.W., D.R.A., C.C., P.L., F.M.P., M.H., W.A.G., and M.C.V.M.) contributed to reviewing and revising the manuscript.

## Ethics Statement

Whole blood samples were collected as part of the NIH FSASD Pilot Natural History Study under the Congenital Disorders of Glycosylation protocol (14‐HG‐0071; NCT04199000), approved by the National Human Genome Research Institute Institutional Review Board.

## Consent

This study was performed in accordance with the ethical standards for medical research outlined in the Helsinki Declaration.

## Conflicts of Interest

The authors declare no conflicts of interest.

## Supporting information


**Figure S1.** Correlation of leukocyte free sialic acid with levels of urinary free sialic acid. A red circle indicates a carrier of two *SLC17A5* missense variants, a green circle represents a carrier of one *SLC17A5* missense and one *SLC17A5* splice site variant, and a blue circle indicates an affected individual with one *SLC17A5* missense and one *SLC17A5* nonsense variant. Data were analyzed using Pearson correlation analysis with r and *p*‐values as indicated.
**Figure S2**. Correlation of leukocyte free sialic acid levels with age in LSD‐negative and LSD cases. Green circles indicate LSD‐negative cases and purple circles represent LSD cases. Data were analyzed using Pearson correlation analysis with *r* and *p*‐values as indicated.
**Figure S3**. Urinary free and total sialic acid levels in FSASD patients compared to age‐dependent reference ranges. Pink circles indicate the reference ranges, while green circles represent individual FSASD patients in relation to these references.
**Figure S4**. Sialic acid levels in unaffected and FSASD human fibroblasts.
(A) Free sialic acid levels, (B) bound sialic acid levels, and (C) total sialic acid levels. The control group includes two unaffected individuals (one replicate per individual). FSASD‐1 corresponds to affected individual #1 from Table 1. SIA‐43 is an individual with intermediate‐severe FSASD, as previously reported by Kleta et al. (PMID: 12794688), and was consented under NIH protocol 76‐HG‐0238. FISAL‐01 and FISAL‐04 are homozygous for the p.Arg39Cys variant, representing mild FSASD. These cell lines were curated by the Biobank Unit of the Finnish Institute for Health and Welfare (THL; Helsinki, Finland) and approved by the THL Institutional Review Board; fibroblasts were then transferred to NIH under a Material Transfer Agreement. GM08497 is also homozygous for the p.Arg39Cys variant and was obtained from the Coriell Institute for Medical Research (https://www.coriell.org/0/Sections/Search/Sample_Detail.aspx?Ref=GM08497&Product=CC). One replicate per cell line. Data were analyzed using an unpaired *t*‐test, with *p*‐values indicated for relevant comparisons.

**Table S1.** Description of subjects in LSD‐negative and LSD cohorts.

## Data Availability

The data supporting the findings of this study are available from the corresponding author upon reasonable request.
