# Supplementary material for: Investigating the Utility of Leukocyte Sialic Acid Measurements in Lysosomal Free Sialic Acid Storage Disorder
Source: JIMD Rep. 2025 Jun 16;66(4):e70029. doi: 10.1002/jmd2.70029 (PMC12171062; doi:10.1002/jmd2.70029)
Supplement: Supplementary file 1 — Figure S1. Correlation of leukocyte free sialic acid with levels of urinary free sialic acid. A red circle indicates a carrier of two SLC17A5 missense variants, a green circle represents a carrier of one SLC17A5 missense and one SLC17A5 splice site variant, and a blue circle indicates an affected individual with one SLC17A5 missense and one SLC17A5 nonsense variant. Data were analyzed using Pearson correlation analysis with r and p‐values as indicated. Figure S2. Correlation of leukocyte free sialic acid levels with age in LSD‐negative and LSD cases. Green circles indicate LSD‐negative cases and purple circles represent LSD cases. Data were analyzed using Pearson correlation analysis with r and p‐values as indicated. Figure S3. Urinary free and total sialic acid levels in FSASD patients compared to age‐dependent reference ranges. Pink circles indicate the reference ranges, while green circles represent individual FSASD patients in relation to these references. Figure S4. Sialic acid levels in unaffected and FSASD human fibroblasts. (A) Free sialic acid levels, (B) bound sialic acid levels, and (C) total sialic acid levels. The control group includes two unaffected individuals (one replicate per individual). FSASD‐1 corresponds to affected individual #1 from Table 1. SIA‐43 is an individual with intermediate‐severe FSASD, as previously reported by Kleta et al. (PMID: 12794688), and was consented under NIH protocol 76‐HG‐0238. FISAL‐01 and FISAL‐04 are homozygous for the p.Arg39Cys variant, representing mild FSASD. These cell lines were curated by the Biobank Unit of the Finnish Institute for Health and Welfare (THL; Helsinki, Finland) and approved by the THL Institutional Review Board; fibroblasts were then transferred to NIH under a Material Transfer Agreement. GM08497 is also homozygous for the p.Arg39Cys variant and was obtained from the Coriell Institute for Medical Research (https://www.coriell.org/0/Sections/Search/Sample_Detail.aspx?Ref=GM08497&Product=CC). On [file JMD2-66-e70029-s001.pdf]

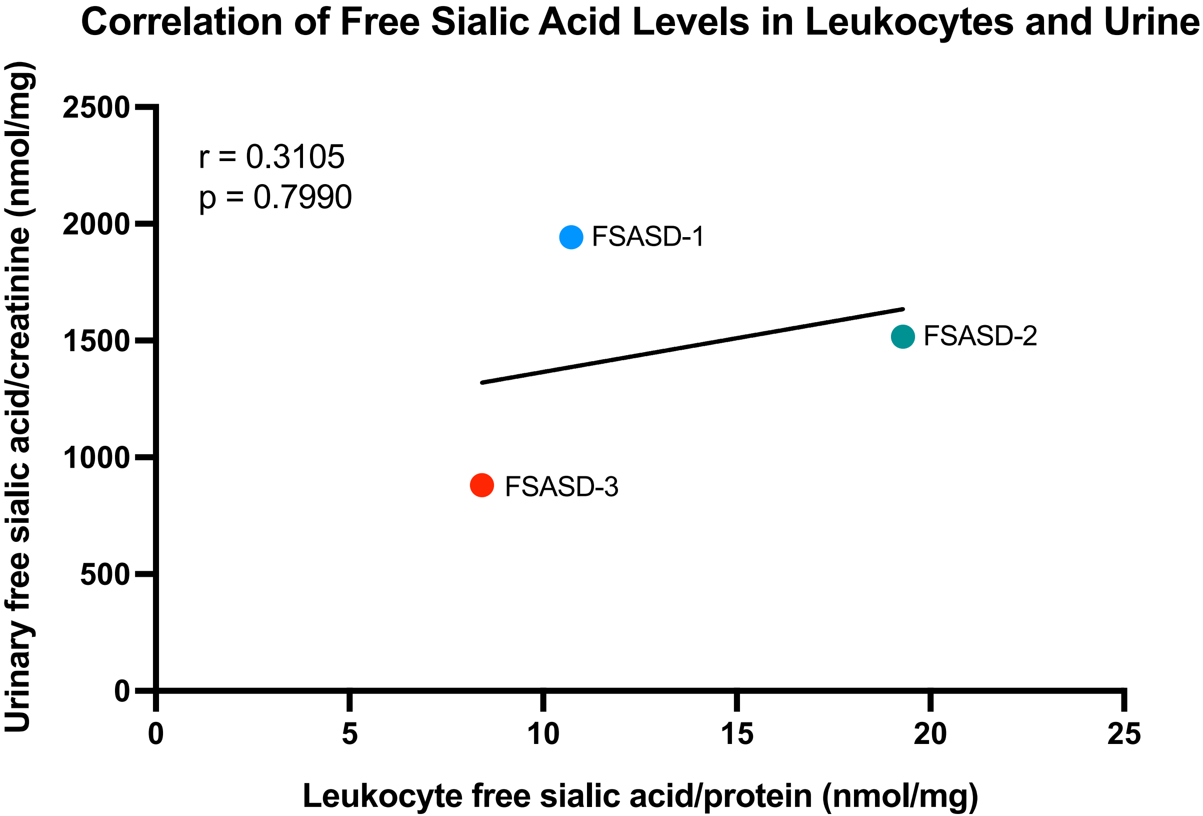
Correlation of Free Sialic Acid Levels in Leukocytes and Urine
Urinary free sialic acid/creatinine (nmol/mg)
2500
2000
1500
1000
500
0
r = 0.3105
p = 0.7990
FSASD-1
FSASD-2
FSASD-3
0
5
10
15
20
25
Leukocyte free sialic acid/protein (nmol/mg)

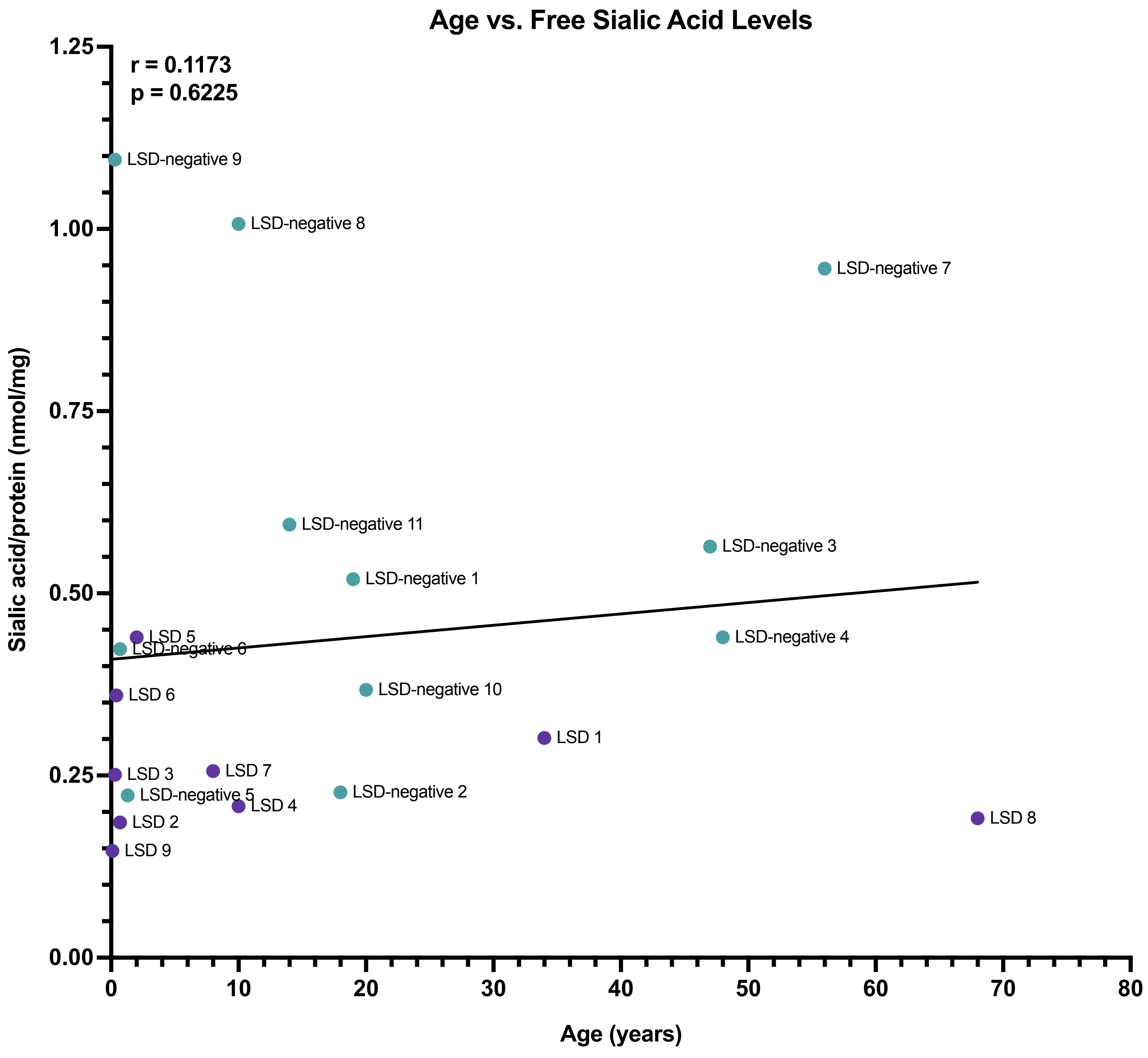
Age vs. Free Sialic Acid Levels
r = 0.1173
p = 0.6225
Sialic acid/protein (nmol/mg)
Age (years)
1.25
1.00
0.75
0.50
0.25
0.00
0
10
20
30
40
50
60
70
80
LSD-negative 9
LSD-negative 8
LSD-negative 7
LSD-negative 11
LSD-negative 3
LSD-negative 1
LSD 5
LSD-negative 6
LSD-negative 4
LSD 6
LSD-negative 10
LSD 1
LSD 3
LSD 7
LSD-negative 5
LSD-negative 2
LSD 4
LSD 2
LSD 8
LSD 9

A

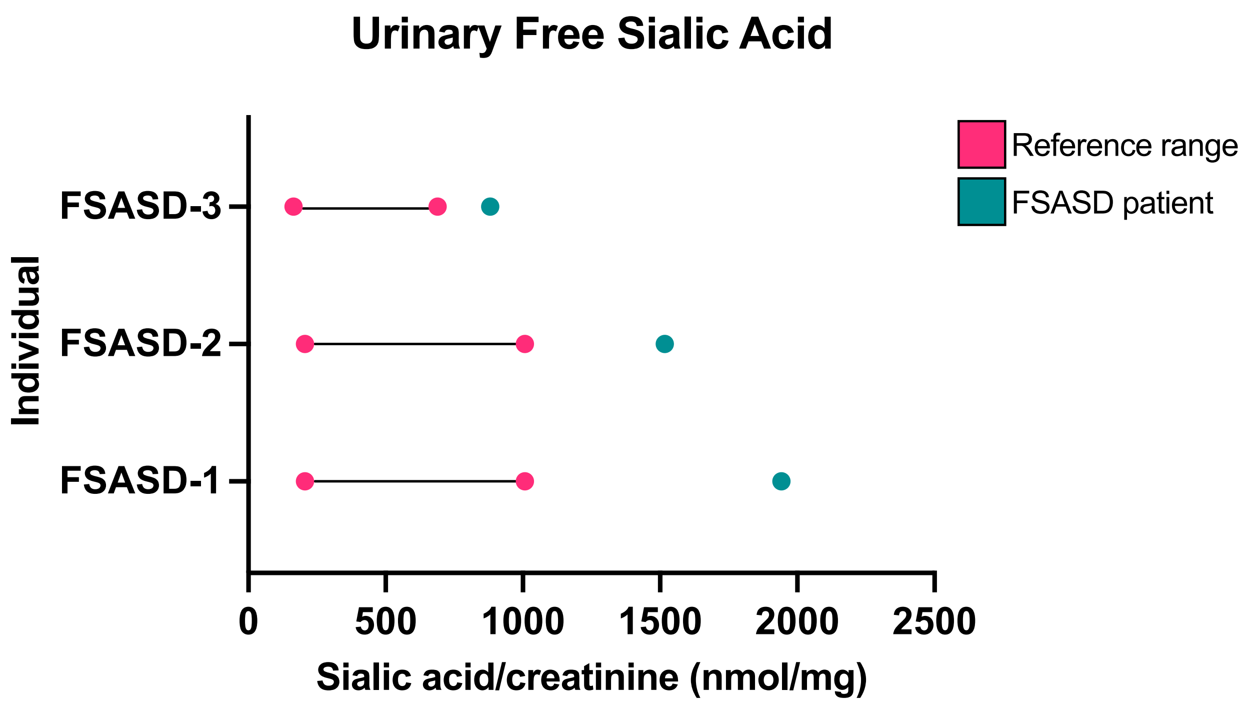
Urinary Free Sialic Acid
Reference range
FSASD patient
FSASD-3
FSASD-2
FSASD-1
Individual
0
500
1000
1500
2000
2500
Sialic acid/creatinine (nmol/mg)

B

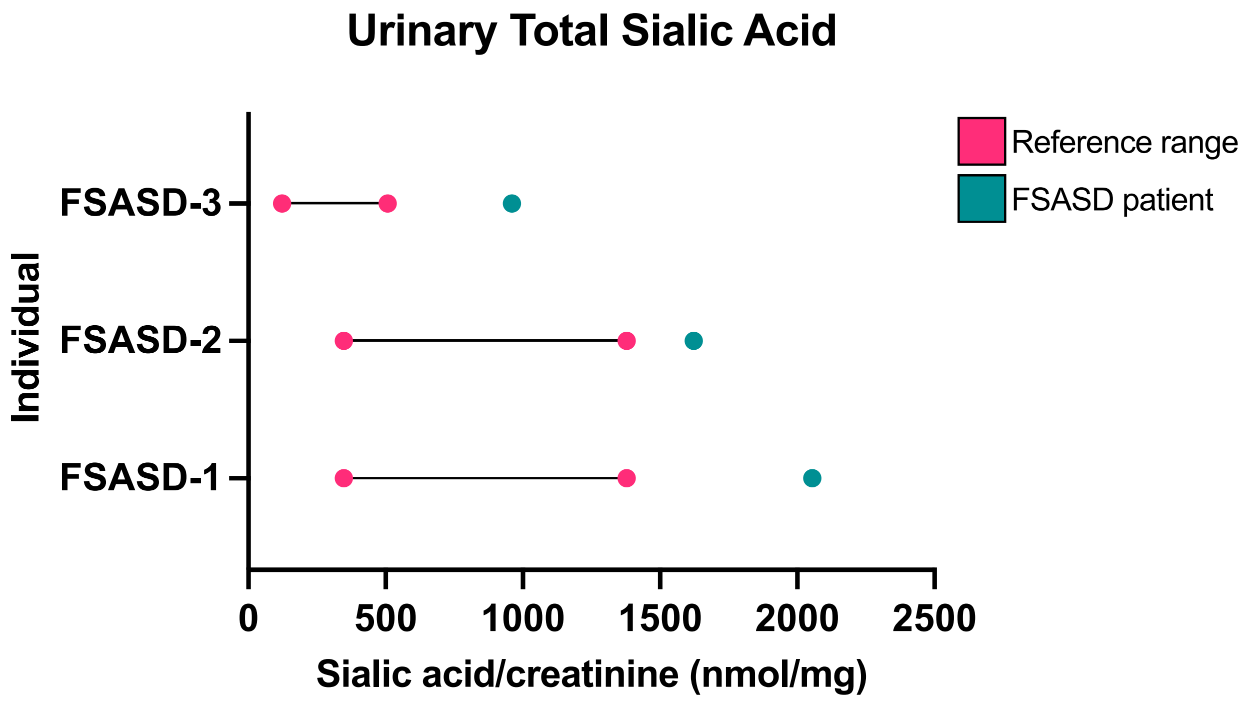
Urinary Total Sialic Acid
Reference range
FSASD patient
FSASD-3
FSASD-2
FSASD-1
Individual
0
500
1000
1500
2000
2500
Sialic acid/creatinine (nmol/mg)

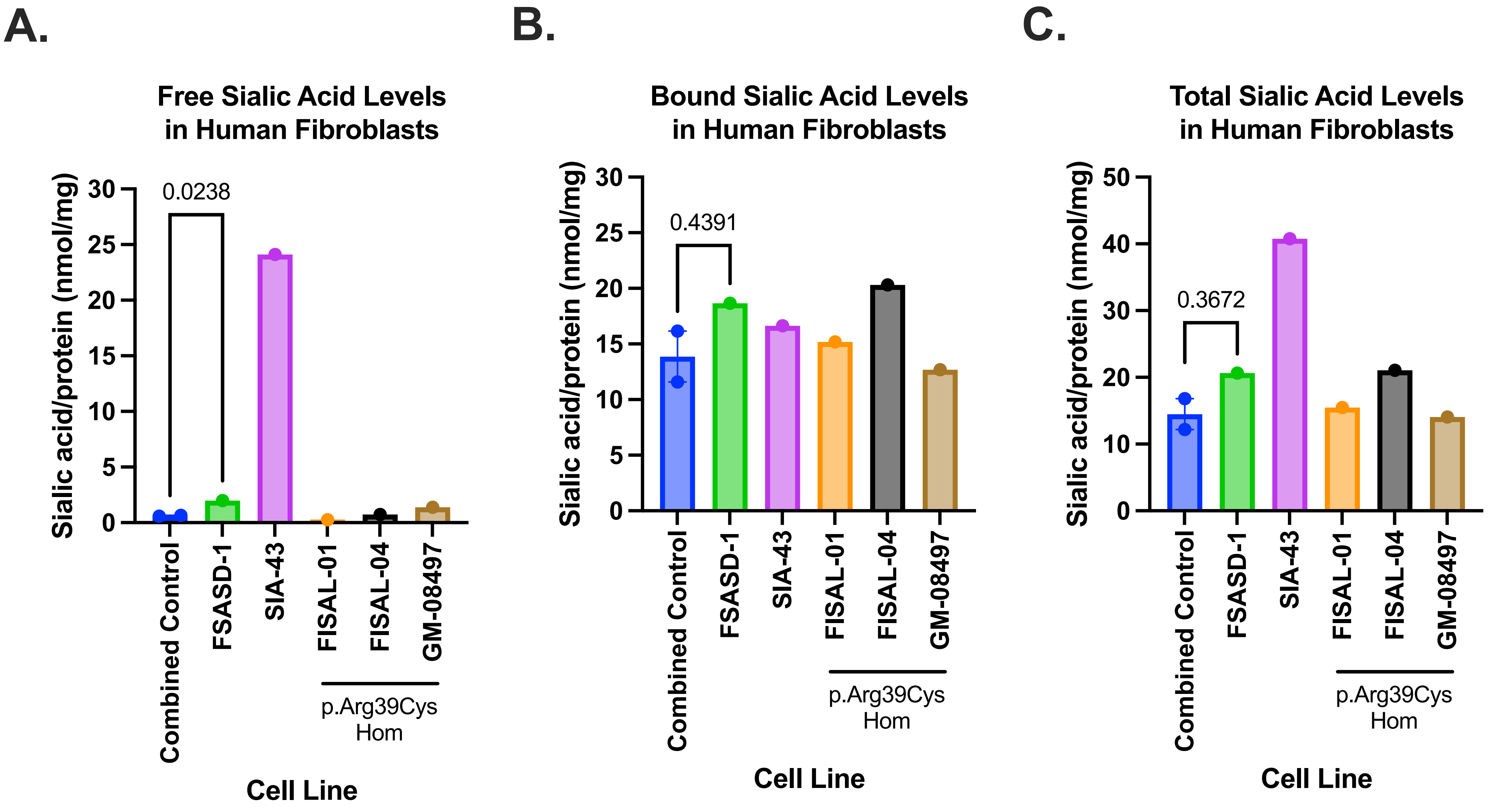

A.
Free Sialic Acid Levels in Human Fibroblasts
Sialic acid/protein (nmol/mg)
0
5
10
15
20
25
30
0.0238
Combined Control
FSASD-1
SIA-43
FISAL-01
FISAL-04
GM-08497
p.Arg39Cys Hom
Cell Line
B.
Bound Sialic Acid Levels in Human Fibroblasts
Sialic acid/protein (nmol/mg)
0
5
10
15
20
25
30
0.4391
Combined Control
FSASD-1
SIA-43
FISAL-01
FISAL-04
GM-08497
p.Arg39Cys Hom
Cell Line
C.
Total Sialic Acid Levels in Human Fibroblasts
Sialic acid/protein (nmol/mg)
0
10
20
30
40
50
0.3672
Combined Control
FSASD-1
SIA-43
FISAL-01
FISAL-04
GM-08497
p.Arg39Cys Hom
Cell Line
