# Supplementary material for: Investigating the Utility of Leukocyte Sialic Acid Measurements in Lysosomal Free Sialic Acid Storage Disorder
Source: JIMD Rep. 2025 Jun 16;66(4):e70029. doi: 10.1002/jmd2.70029 (PMC12171062; doi:10.1002/jmd2.70029)
Supplement: Supplementary file 2 — Table S1. Description of subjects in LSD‐negative and LSD cohorts. [file JMD2-66-e70029-s002.docx]

| **Supplementary Table 1.** Description of subjects in LSD-negative and LSD cohorts | | | | | |  |
| --- | --- | --- | --- | --- | --- | --- |
| **Group** | **Subject ID** | **Diagnosis** | **Age at sample collection (years)** | **Sex** | **Testing performed for diagnosis** |  |
| LSD-negative | 24-23868 | LSD-negative | 19 | M | N/A |  |
|  | 24-23871 | LSD-negative | 18 | F | N/A |  |
|  | 24-23469 | LSD-negative | 47 | F | N/A |  |
|  | 24-23465 | LSD-negative | 48 | F | N/A |  |
|  | 24-23422 | LSD-negative | 1.3 | F | N/A |  |
|  | 24-23332 | LSD-negative | 0.7 | F | N/A |  |
|  | 24-23329 | LSD-negative | 56 | M | N/A |  |
|  | 24-23252 | LSD-negative | 10 | M | N/A |  |
|  | 24-23288 | LSD-negative | 0.3 | M | N/A |  |
|  | 24-23262 | LSD-negative | 20 | F | N/A |  |
|  | 24-23218 | LSD-negative | 14 | M | N/A |  |
| LSD | 24-03070 | Fabry disease | 34 | M | Alpha-galactosidase |  |
|  | 24-01681 | Mucopolysaccharidosis type I | 0.7 | F | Alpha-iduronidase |  |
|  | 24-02622 | GM1 gangliosidosis | 0.3 | F | Beta-galactosidase |  |
|  | 24-20917 | Mucopolysaccharidosis type IIIC | 10 | F | Acetyl CoA: glucosamine N-acetyltransferase |  |
|  | 24-24285 | Mucopolysaccharidosis type IIIA | 2 | M | Heparan-N-sulfatase |  |
|  | 24-23221 | Metachromatic leukodystrophy | 0.4 | F | Arylsulfatase A |  |
|  | 24-01680 | Alpha-mannosidosis | 8 | M | Alpha-mannosidase |  |
|  | 23-19769 | Gaucher disease | 68 | M | Beta-glucosidase |  |
|  | 24-03686 | Late-onset Pompe disease | 0.1 | F | Alpha-glucosidase |  |
| Abbreviations include: LSD, lysosomal storage disorders; F, female; M, male; N/A, not applicable. | | | | | |  |
|  |  |  |  |  |  |  |
